# Study of the Prevalence of Atypical Scheuermann's Kyphosis Using Computed Tomography Scans

**DOI:** 10.1055/s-0044-1792119

**Published:** 2024-12-21

**Authors:** Gabriel de Valentim Souza, Lucas Yuya Oki, Alexandre Martins Portelinha, Mariana Demétrio de Sousa Pontes, Carlos Fernando Pereira da Silva Herrero

**Affiliations:** 1 Faculdade de Medicina de Ribeirão Preto, Universidade de São Paulo, Ribeirão Preto, SP, Brasil; 2 Divisão de Ortopedia Pediátrica, Hospital São Lucas, Ribeirão Preto, SP, Brasil; 3 Departamento de Ortopedista e Traumatologia, Hospital das Clínicas da Faculdade de Medicina de Ribeirão Preto, Ribeirão Preto, SP, Brasil; 4 Departamento de Ortopedia e Anestesiologia, Hospital das Clínicas da Faculdade de Medicina de Ribeirão Preto, Universidade de São Paulo, Ribeirão Preto, SP, Brasil

**Keywords:** abdomen, adolescent, prevalence, Scheuermann disease, scoliosis

## Abstract

**Objective** The aim of the present study was to detect atypical Scheuermann's disease through computed tomography scans and estimate its prevalence.

**Methods** This cross-sectional observational study involved 1,287 computed tomography scans from patients aged 18 to 40 years of both genders. The criteria for diagnosing atypical Scheuermann's disease included wedging of 5° in 3 consecutive vertebrae, combined with a total Cobb angle of 10° or more within the thoracolumbar interval from T8 to L2. Positive cases were assessed for kyphosis severity, presence of Schmorl's nodes, and scoliosis. Prevalence estimation and correlation analysis with age and sex were performed.

**Results** The study identified 28 cases of atypical Scheuermann's kyphosis, indicating a prevalence of 2.8%.

**Conclusion** The current research, utilizing abdominal tomography, offers valuable insights into the prevalence of Scheuermann's disease in its atypical form within the sampled population.

## Introduction

Scheuermann's disease (SD) was defined by Holger Werfel Scheuermann in 1920 and is also known as osteochondritis juvenile dorsi or kyphosis dorsalis juvenilis. 1 Sorensen described the radiographic criteria for typical SD, including anterior wedging greater than 5° in at least 3 adjacent vertebral bodies. 2

Schmorl's nodes, irregular and flattened vertebral endplates, narrowed intervertebral disc spaces, and anteroposterior elongation of the apical vertebral bodies are other associated radiological features of SD. 2 3 4 5 6 The affection leads to rigid kyphosis of the median and lower thoracic, or upper lumbar regions. 7

Two patterns have been defined in SD based on the affected area of the vertebra. 7 8

In the typical or classic form, the thoracic region is frequently affected and is characterized by thoracic kyphosis increase and wedging of the vertebral bodies. This pattern is also accompanied by nonstructural hyperlordosis of the cervical and lumbar spine. 7 9 10 11 On the other hand, SD of atypical pattern (thoracolumbar or lumbar) has been defined later and is distinguished from the typical one by lack of thoracic kyphosis and evident wedging of the vertebrae. 4 12 13 Also, the apex of the kyphosis is located at the thoracolumbar junction (T11–T12) in an atypical pattern of the disease. 4 Such pathologies account for 25 to 80% of SD. 4

Despite the importance of SD, knowledge of its epidemiology needs to be improved. Most articles mention that the prevalence varies from 0.4 to 8.0%, 2 7 14 and a few recent studies have examined the prevalence of SD, but only in individuals aged from either 45 or 50 to 80 years or older, with a reported prevalence of 4.0 to 8.0%. 15 Besides, these studies are contradictory regarding the prevalence between the genders. Some studies have suggested that the prevalence in men may be higher than in women, 3 8 16 17 18 19 while other publications suggest that it involves each gender equally. 15 20

Furthermore, previous studies on the prevalence of SD did not evaluate the atypical pattern of the condition separately. 7 15 20

Thus, the present study aimed to determine the prevalence of atypical SD in patients aged between 18 and 40 years using abdomen tomography as a screening tool.

## Material and Methods

### Study Design and Patient Population

This is a cross-sectional study. The hospital's Ethics Committee and Internal Review Board approved the study protocol before initiation under number (CAAE: 51849821.6.0000.5440). The study design was based on evaluating tomographic images of the abdomen. The exclusion criteria used in the present study were patients aged < 18 years old or > 40 years old, with previous spine surgery, presence of thoracolumbar spine diseases such as fractures, tumors, infections, ankylosing spondylitis, and familial hyperostotic disease. The inclusion criteria were patients aged between 18 and 40 years old and the presence of adequate images for analysis according to previously established parameters.

Retrospectively, we selected abdomen tomographic images of 1,287 individuals (756 females and 531 males) obtained from a database of patients referred to a radiologic private clinic from October 2021 to August 2022.

### Computed Tomography (CT) Acquisition

Imaging exams were performed using a GE LightSpeed VCT device. The CT images obtained were reconstructed with 1.25-mm thick slices for the axial plane. The reading and reproduction of the images, as well as their reconstructions, were carried out using the Carestream PACS software system, version 12.1.5.1156 (Carestream Health, Rochester, NY, USA).

### Image Analysis

**Fig. 1:**
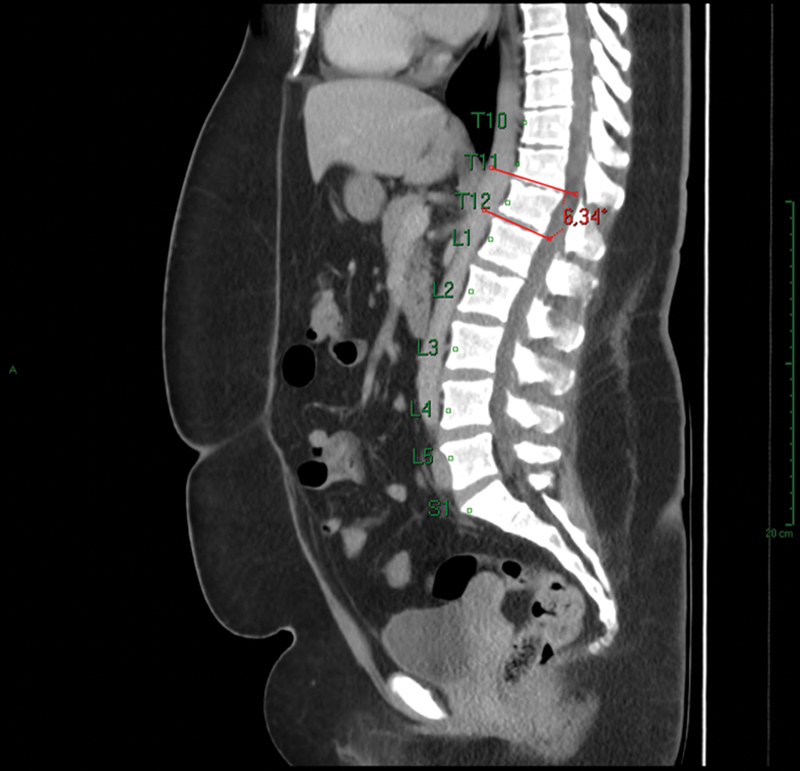
Individual Cobb angle measurement in vertebra with wedging.

The parameter to identify patients with atypical SD was wedging in 3 consecutive vertebrae by 5° or more (measurement of the Cobb angle in each vertebra), with a total Cobb angle of 10° or higher in the sagittal-plane tomographic images. The thoracolumbar segment considered in our study was the one from T8 to L2 (thoracolumbar transition) ( Figs. 1 2 ).

**Fig. 2:**
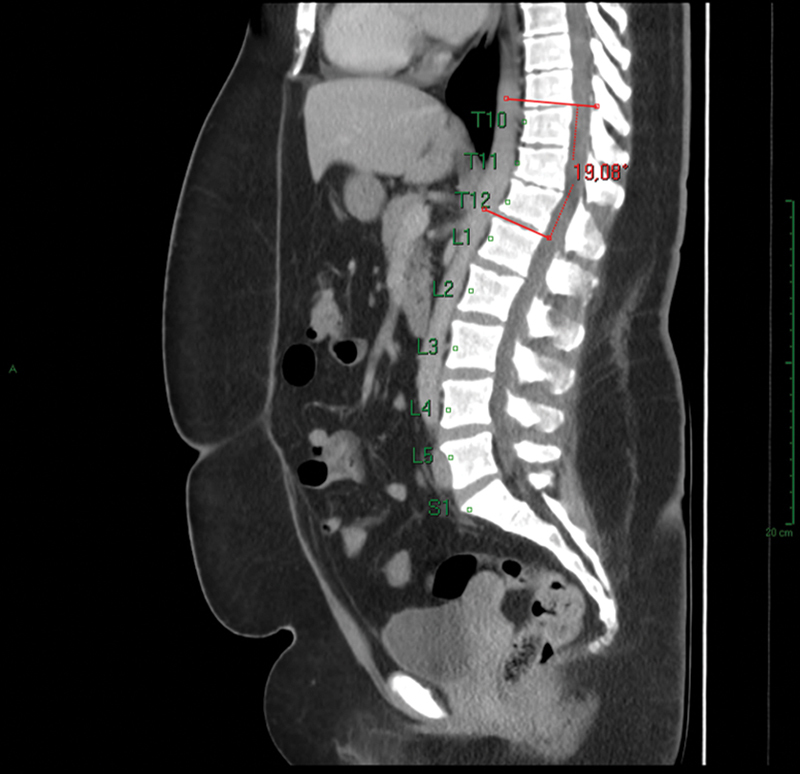
Total Cobb angle measurement after identifying wedging greater than 5° in 3 consecutive vertebrae (T10–T12).

**Fig. 3:**
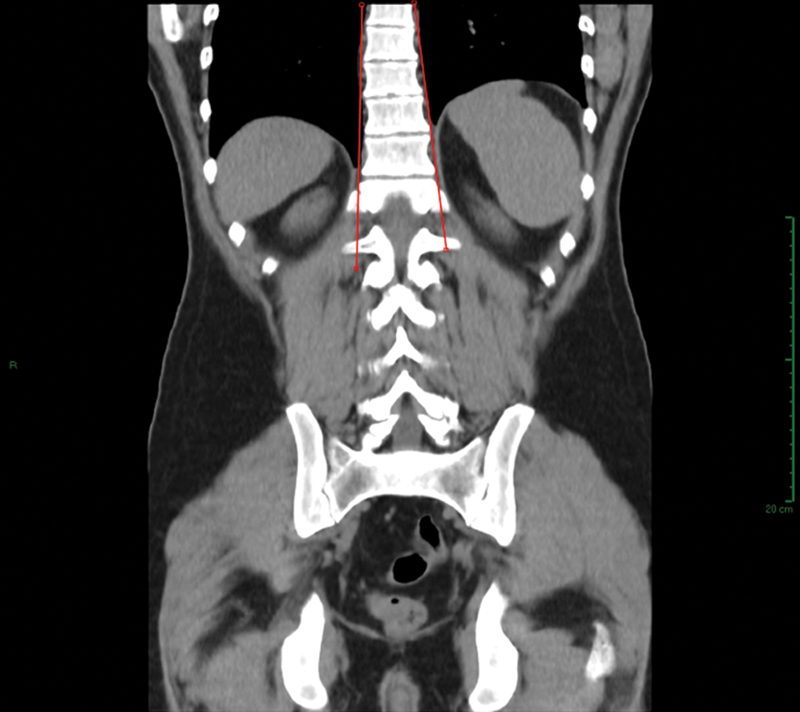
Coronal slice used for diagnosing scoliosis in positive cases of atypical Scheuermann's disease, as present in the above-mentioned case.

As coronal image visualization was available in all the exams evaluated, in which it was possible to observe the spine from the sacrum until the thoracic segment, it was also possible to evaluate the presence or absence of scoliosis in patients. Therefore, scoliosis was investigated using the CT coronal images. The method used for this was the evaluation of the coronal alignment between the cervical, thoracic, and lumbar vertebrae and the Cobb angle measurement ( Fig. 3 ).

**Fig. 4:**
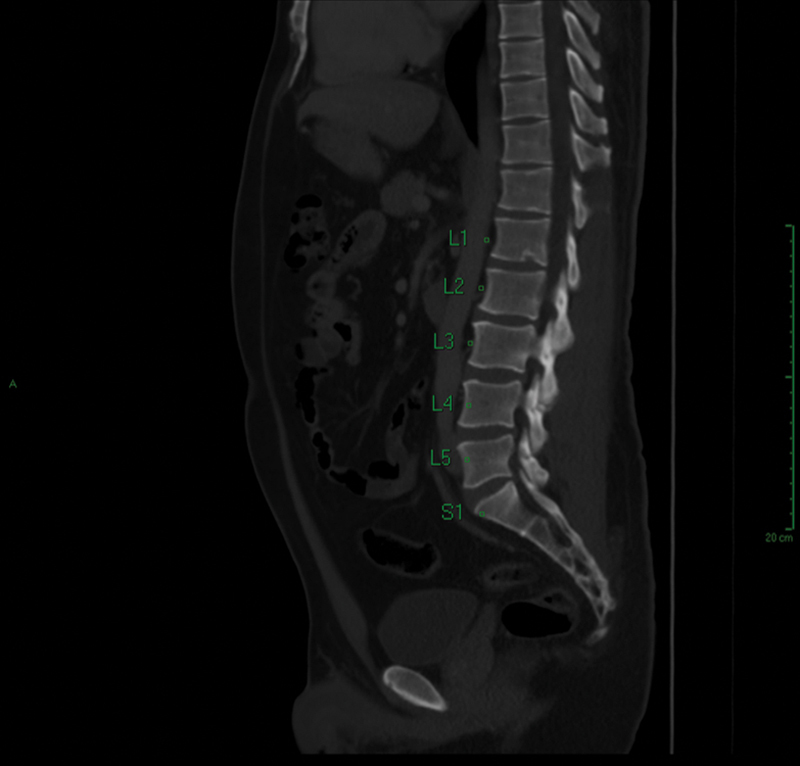
Sagittal cut used for diagnosing Schmorl's nodes (at the level of L1) in positive cases of the disease.

In cases classified as positive for atypical SD, the level of kyphosis and the presence of Schmorl's nodules and scoliosis were also assessed. These patients were identified according to sex and age to correlate the tomographic findings ( Fig. 4 ). Data collection, through analysis of image exams, was carried out by a team composed of 3 researchers using the Carestream PACS system software version 12.1.5.1156..

### Statistical Analysis

The data were compiled into a Microsoft Excel software spreadsheet (Microsoft Corp., Redmond, WA, USA) and analyzed using the R software version 4.2 (R Foundation for Statistical Computing, Vienna, Austria). Continuous data were expressed as mean, standard deviation, median, and interquartile range (IQR), and categorical data were described as absolute values and percentages.

The T-test or Yuen-Welch robust test was used to compare the mean between the two groups, depending on the variable's distribution. Similarly, to compare three or more groups of continuous data, the analysis of variance (ANOVA) test or the robust one-way ANOVA test was used, with a trim of 0.2, depending on the distribution of the variable. In situations of *p* < 0.05, multiple paired tests were performed with *p* -value adjustment using the Bejanmini-Yuketieli method, to verify which groups had a significant difference.

The Shapiro-Francia test was used to test the distribution of variables. To compare categorical variables, with only two groups, the Fisher or Chi-squared test was used, according to the following rule: if more than 20% of the expected values of the counting cells were greater than 5, the Fisher test was used, otherwise the Chi-squared test was used (Kim, 2017). 26 In the case of categorical variables with more than 3 levels, the Chi-squared test was used. Values of *p* < 0.05 were considered statistically significant.

## Results

Tomographic images from 1,287 patients were randomly selected, with a median age of 33 (IQR: 5) years old (18–40 years old), 756 (58.8%) male and 531 (41.2%) female patients. The median age among the men was 33 (IQR: 6) years old (19–40 years old), and among the women, it was 33 (IQR: 10) years old (19–40 years old). There was no excluded patient. Out of the 1,287 CT scans evaluated, 28 patients were diagnosed with atypical SD, representing 2.2% of the total patients included in this study.

### Age

**Fig. 5:**
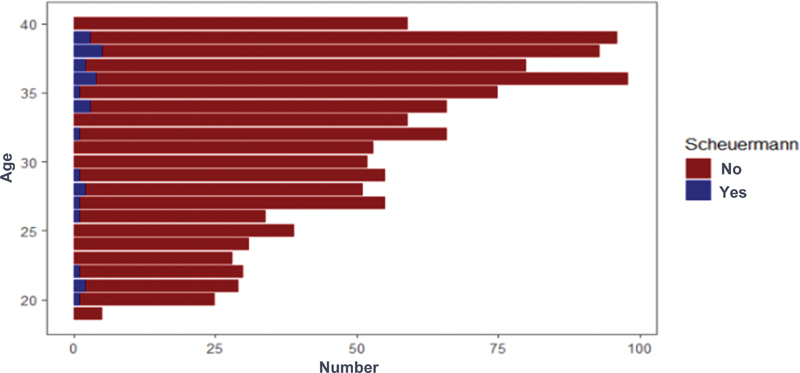
Prevalence of atypical Scheuermann's disease according to age.

Regarding patient age, younger patients (18–29 years old) presented higher Cobb values than older patients (30–40 years old), with an average of 17.4° and 15°, respectively ( Fig. 5 ). However, when evaluated by the Spearman correlation test, the groups had no significant difference ( *p* = 0.28).

### Gender

**Fig. 6:**
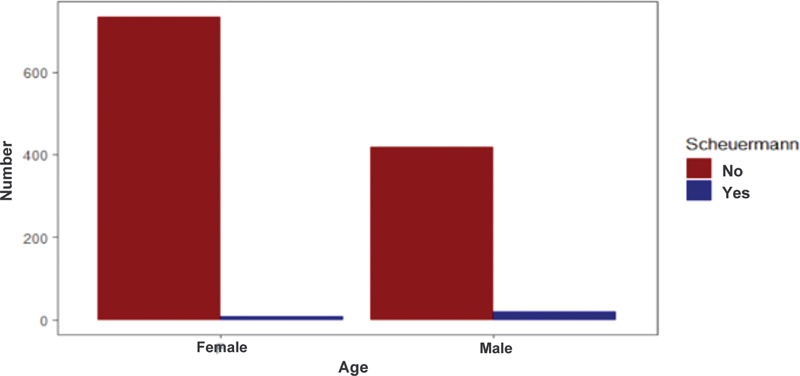
Prevalence of atypical Scheuermann's disease according to gender.

When differentiating regarding gender, a significant difference was observed, with a higher prevalence of atypical SD in male patients, representing 4.7% of the condition among men and 1.1% among women ( *p* < 0.01) ( Fig. 6 ).

### Spine Level

**Fig. 7:**
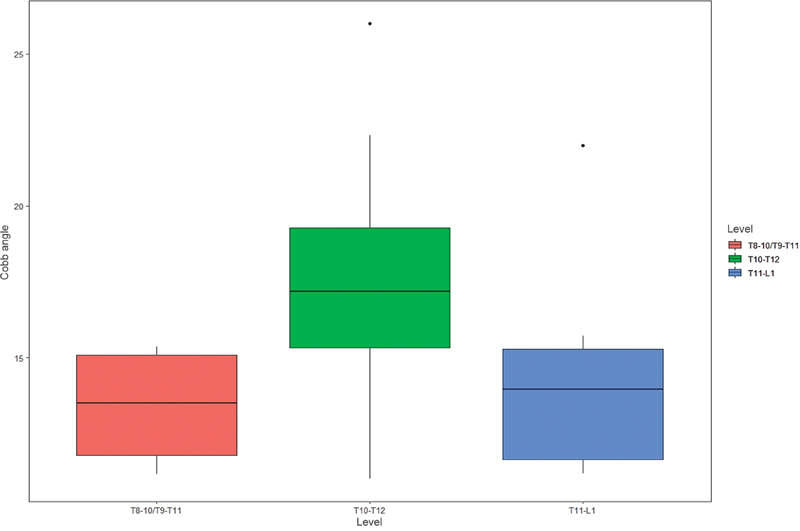
Prevalence of atypical Scheuermann's disease according to the level of spinal involvement.

When stratifying the levels of spinal involvement in atypical SD ( Fig. 7 ), the T10-to-T12 level was the most prevalent one, representing 50% (n = 14) of the total positive cases. For conducting the one-way ANOVA test and comparing multiple groups with the linear contrasts test, the T9-to-T11 and the T8-to-T10 interval groups were combined into a single group (T8–10/T9–T11). It was observed that there was a significant difference in the total Cobb angle value between the 3 groups ( *p* = 0.02, effect size = 0.68 [0.21–1.58]). Moreover, when the linear contrasts test was performed, a significant difference was found between the T10-to-T12 and the T11-to-L1 groups ( *p* = 0.046). In contrast, no significant difference was found when comparing other groups.

### Scoliosis

**Fig. 8:**
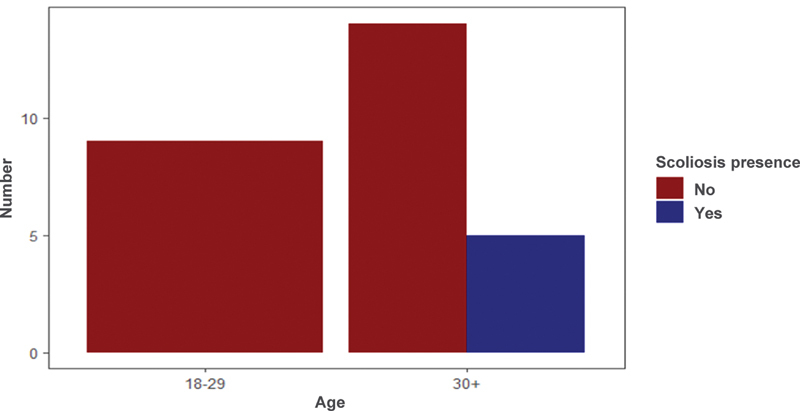
Comparison of scoliosis presence and patient sex in atypical Scheuermann's disease.

When comparing the presence of scoliosis in patients with atypical SD according to gender, there was no significant difference. Scoliosis among women was found in 14% of the patients and 25% among the male patients (Fisher's exact test; *p* = 1) ( Fig. 8 ).

### Schmorl Nodes

**Fig. 9:**
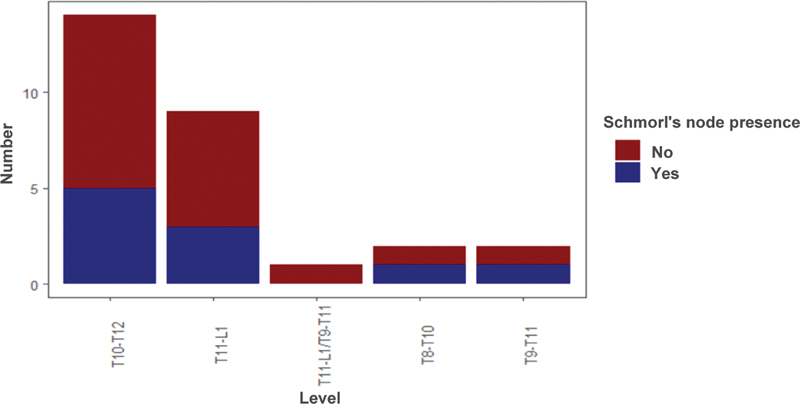
Presence of Schmorl's nodes in patients with atypical Scheuermann's disease.

Of the 28 patients with atypical SD, 10 (37%) presented Schmorl's nodules on tomographic examination ( Fig. 9 ). No association was found between age groups or gender and the presence of Schmorl's nodules ( *p* = 1).

## Discussion

In this epidemiological study of the Brazilian population, we found a prevalence of atypical SD of 2.8%, utilizing the criteria defined by Sorensen. 2

Since there is no uniform standard for the radiological diagnosis of the atypical form of the disease, we chose to use the Sorensen criteria as it is the standard most used in other previous research. 15 20

The design of our study based on CT allowed us to identify atypical SD in patients without clinical complaints, which would otherwise be missed in a study based on the patient's clinical complaints. As the demand for the tomography exam was due to complaints other than the spine, we reduced the presence of selection bias in our sample since the selection of patients from a spine surgery office would include a selection criterion, that is complaining of deformity or spine pain.

Typically, patients present Scheuermann's kyphosis during the bone growth phase of adolescence. 5 7 8 Thus, all patients presenting the radiological characteristics of the disease already had it at the time of our evaluation. Furthermore, to avoid the possibility of false positive cases resulting from degenerative disc disease, we limited the age criteria for including patients in our study. Therefore, none of the cases diagnosed with atypical SD may correspond to patients with degenerative disc disease, and we have not failed to identify any patient who has not yet developed the disease.

There is still controversy in the literature regarding the prevalence of SD according to patients' gender. Thus, some studies showed a similar prevalence in both genders, 3 15 20 21 22 23 but most previous research demonstrated a higher incidence in men. 1 3 8 17

However, none of these studies evaluated separately patients diagnosed with atypical SD. Thus, despite agreeing with most studies, our results with a higher prevalence of SD in males only represent patients with the atypical form of the disease.

Sorensen described radiographic criteria for typical SD, including anterior wedging greater than 5° in at least 3 adjacent vertebral bodies. 2 Schmorl's nodes, irregular vertebral endplates, narrowed intervertebral disc, and anteroposterior elongation of the apical vertebral bodies are other radiological features associated with MS. On the other hand, Blumenthal et al. 24 described the criteria for atypical MS, including wedging in one or two vertebral bodies, changes in the vertebral endplate, narrowing of the disc space, and anterior Schmorl's nodes.

We chose to use Sorensen's criterion of anterior vertebral wedging of more than 5° because it is a more objective criterion than simply using vertebral wedging as suggested by Blumenthal et al. 24 We were also able to investigate the presence of scoliosis and Schmorl's nodules, and just as previous studies have shown, the prevalence of scoliosis was around a third of cases. 25 In around 40% of the exams, we could identify the presence of Schmorl's nodules, which agree with the findings of Heithoff et al., 12 according to which the presence of Schmorl's nodules would be just one of the diagnostic criteria for atypical DS.

The main limitation of the present study lies in the fact that we used the CT exam to identify the presence of SD since the exam of choice is the full spine X-ray. However, as the diagnosis of atypical SD is based on the individual wedging of each vertebra, the chosen exam did not affect our results. Furthermore, we reduced the possibility of selection bias since patients underwent the examination for reasons other than the spine.

## Conclusion

Scheuermann's kyphosis is a frequent spinal deformity. Our study, obtained from a large convenience sample of patients evaluated with abdomen tomography, allows a better understanding of the actual prevalence of the atypical pattern of this condition. The CT examination made it possible to study a population sample without selection bias and to find a prevalence of 2.8% of atypical SD in the selected sample.
