# Supplementary material for: Study of the Prevalence of Atypical Scheuermann's Kyphosis Using Computed Tomography Scans
Source: Rev Bras Ortop (Sao Paulo). 2024 Dec 21;59(6):e854–60. doi: 10.1055/s-0044-1792119 (PMC11663052; doi:10.1055/s-0044-1792119)
Supplement: Related texts

# Research article: Estudo de prevalência da cifose atípica de Scheuermann por meio de tomografias computadorizadas

**Keywords:** abdome, adolescente, doença de Scheuermann, escoliose, prevalência

## Abstract

**Objetivo** O objetivo deste estudo foi identificar a doença de Scheuermann (DS) atípica por meio de tomografias computadorizadas (TCs) e estimar a prevalência desta afecção.

**Métodos** Estudo observacional transversal com inclusão de 1.287 TCs de pacientes de ambos os sexos, com faixa etária entre 18 e 40 anos. Os critérios utilizados para identificação da DS atípica foi a formação de cunha de 5° em 3 vértebras consecutivas e o ângulo de Cobb total maior ou igual a 10° no intervalo toracolombar entre T8 e L2. Nos casos positivos, foi avaliado o nível de cifose e a presença de nódulos de Schmorl e escoliose. A prevalência da doença foi estimada e a correlação à idade e ao sexo dos pacientes foi realizada.

**Resultados** Foram encontrados 28 casos de cifose atípica de Scheuermann, com prevalência de 2,8%.

**Conclusão** Esta pesquisa, utilizando tomografia abdominal, traz informações valiosas sobre a prevalência da DS em sua forma atípica na população avaliada.

## Introdução

A doença de Scheuermann (DS) foi definida por Holger Werfel Scheuermann em 1920 e é também conhecida como osteocondrite juvenil do dorso ou cifose dorsal juvenil. 1 Sorensen descreveu os critérios radiográficos da DS típica, incluindo cunha anterior maior que 5° em pelo menos 3 corpos vertebrais adjacentes. 2

Nódulos de Schmorl, irregularidade e achatamento das placas terminais vertebrais, estreitamento dos espaços dos discos intervertebrais e alongamento anteroposterior dos corpos vertebrais apicais são outras características radiológicas associadas à DS. 2 3 4 5 6 A doença leva à cifose rígida da região torácica mediana ou inferior ou lombar superior. 7

Há dois padrões definidos de DS com base na área acometida da vértebra. 7 8

A forma típica, ou clássica, geralmente afeta a região torácica e é caracterizada por aumento da cifose torácica e formação de cunha nos corpos vertebrais. Este padrão também é acompanhado por hiperlordose não estrutural da coluna cervical e lombar. 7 9 10 11 Por outro lado, a DS de padrão atípico (toracolombar ou lombar) foi definida depois e se distingue da típica pela ausência de cifose torácica e evidente formação de cunhas vertebrais. 4 12 13 Além disso, o ápice da cifose está localizado na junção toracolombar (T11–T12) no padrão atípico de doença. 4 Essas patologias são responsáveis por 25 a 80% dos casos de DS. 4

Apesar da importância da DS, o conhecimento de sua epidemiologia precisa aumentar. A maioria dos artigos menciona que a prevalência varia de 0,4 a 8,0%, 2 7 14 e poucos estudos recentes examinaram esta prevalência apenas em indivíduos com idade entre 45 ou 50 a 80 anos ou mais, relatando-a em 4,0 a 8,0%. 15 Além disso, os estudos são contraditórios quanto à prevalência entre os sexos. Alguns estudos sugeriram que a prevalência em homens pode ser maior do que em mulheres, 3 8 16 17 18 19 enquanto outras publicações sugerem prevalências iguais. 15 20

Ademais, estudos anteriores sobre a prevalência da DS não avaliaram o padrão atípico da doença separadamente. 7 15 20

Assim, este estudo teve como objetivo determinar a prevalência da DS atípica em pacientes com idade entre 18 e 40 anos utilizando a tomografia de abdômen como ferramenta de triagem.

## Materiais e Métodos

## Delineamento Experimental e População do Estudo

Este é um estudo transversal. O Comitê de Ética do hospital e o Comitê de Ética em Pesquisa aprovaram o protocolo do estudo antes de seu início sob número CAAE: 51849821.6.0000.5440. O delineamento experimental foi baseado na avaliação de imagens tomográficas do abdômen. Os critérios de exclusão utilizados foram idade < 18 anos ou > 40 anos, cirurgia prévia da coluna, presença de doenças da coluna toracolombar, como fraturas, tumores, infecções, espondilite anquilosante e doença hiperostótica familiar. Os critérios de inclusão foram idade entre 18 e 40 anos e presença de imagens adequadas para análise de acordo com parâmetros previamente estabelecidos.

Retrospectivamente, selecionamos imagens tomográficas de abdômende 1.287 indivíduos (756 mulheres e 531 homens) obtidas de um banco de dados de pacientes encaminhados a uma clínica radiológica privada de outubro de 2021 a agosto de 2022.

## Aquisição de Tomografias Computadorizadas (TCs)

Os exames de imagem foram realizados com o aparelho GE LightSpeed VCT. As imagens de TC obtidas foram reconstruídas com cortes de 1,25 mm de espessura em plano axial. A leitura, reprodução e reconstrução das imagens foram realizadas com o *software* Carestream PACS, versão 12.1.5.1156 (Carestream Health, Rochester, NY, EUA).

## Análise das Imagens

O parâmetro para identificação de pacientes com DS atípica foi a formação de cunha em 3 vértebras consecutivas de 5° ou mais (medida do ângulo de Cobb em cada vértebra) e ângulo de Cobb total de 10° ou mais nas imagens tomográficas em plano sagital. O segmento toracolombar considerado em nosso estudo foi de T8 a L2 (transição toracolombar) ( Figs. 1 e 2 ).

**Fig. 1:** Medida individual do ângulo de Cobb em vértebra com cunha.

**Fig. 2:** Medida do ângulo total de Cobb após identificação de formação de cunha maior que 5° em 3 vértebras consecutivas (T10–T12).

Como todos os exames avaliados tinham vistas coronais, permitindo a observação da coluna vertebral desde o sacro até o segmento torácico, também foi possível avaliar a presença ou ausência de escoliose nos pacientes. Portanto, a escoliose foi investigada usando as imagens coronais de TC. O método utilizado para isso foi a avaliação do alinhamento coronal entre as vértebras cervicais, torácicas e lombares e a medida do ângulo de Cobb ( Fig. 3 ).

**Fig. 3:** Corte coronal usado para diagnóstico de escoliose em casos positivos de doença de Scheuermann atípica, como presente no caso acima.

Nos casos classificados como positivos para DS atípica, também foram avaliados o nível de cifose e a presença de nódulos de Schmorl e escoliose. Esses pacientes foram identificados de acordo com o sexo e a idade para correlação com os achados tomográficos ( Fig. 4 ). A coleta de dados, por meio da análise de exames de imagem, foi realizada por uma equipe composta por três pesquisadores utilizando o *software* do sistema Carestream PACS versão 12.1.5.1156.

**Fig. 4:** Corte sagital utilizado para diagnóstico de nódulos de Schmorl (em nível de L1) em casos positivos da doença.

## Análise Estatística

Os dados foram compilados em uma planilha do *software* Microsoft Excel (Microsoft Corp., Redmond, WA, EUA) e analisados usando o *software* R versão 4.2 (R Foundation for Statistical Computing, Viena, Áustria). Os dados contínuos foram expressos como média, desvio-padrão, mediana e intervalo interquartil (IIQ) e os dados categóricos foram descritos como valores absolutos e porcentagens.

O teste T ou teste robusto de Yuen-Welch foi utilizado para comparação da média entre dois grupos dependendo da distribuição da variável. Da mesma forma, para comparação de três ou mais grupos de dados contínuos, foi utilizado o teste análise de variância (ANOVA) ou o teste ANOVA robusto unidirecional com corte de 0,2 dependendo da distribuição da variável. Caso *p* fosse menor que 0,05, foram realizados testes múltiplos pareados com ajuste do valor de *p* pelo método de Bejanmini-Yuketieli para determinação dos grupos com diferença significativa.

O teste de Shapiro-Francia testou a distribuição das variáveis. Para comparar variáveis categóricas, com apenas 2 grupos, foi utilizado o teste de Fisher ou qui-quadrado, de acordo com a seguinte regra: se mais de 20% dos valores esperados das células de contagem fossem maiores que 5, o teste de Fisher era utilizado; caso contrário, o teste qui-quadrado era usado (Kim, 2017). 26 No caso de variáveis categóricas com mais de 3 níveis, foi utilizado o teste qui-quadrado. Valores de *p* < 0,05 foram considerados estatisticamente significativos.

## Resultados

Imagens tomográficas de 1.287 pacientes, com idade mediana de 33 (IIQ, 5) anos (18–40 anos) foram selecionadas aleatoriamente. Dentre os pacientes, 756 (58,8%) eram homens e 531 (41,2%), mulheres. A idade mediana entre os homens foi de 33 (IIQ, 6) anos (19–40 anos) e, entre as mulheres, 33 (IIQ, 10) anos (19–40 anos). Nenhum paciente foi excluído. Das 1.287 TCs, 28 pacientes foram diagnosticados com DS atípica, representando 2,2% do total de indivíduos incluídos neste estudo.

## Idade

Em relação à idade dos pacientes, os mais jovens (18–29 anos) apresentaram valores de Cobb maiores do que os mais velhos (30–40 anos), com média de 17,4° e 15°, respectivamente. ( Fig. 5 ). Entretanto, quando avaliados pelo teste de correlação de Spearman, não houve diferença estatística entre os grupos ( *p* = 0,28).

**Fig. 5:** Prevalência da doença de Scheuermann atípica por idade.

## Sexo

Na diferenciação por gênero, observou-se diferença significativa, com maior prevalência de DS atípica em pacientes do sexo masculino, representando 4,7% dos casos entre homens e 1,1% entre amulheres ( *p* < 0,01) ( Fig. 6 ).

**Fig. 6:** Prevalência da doença de Scheuermann atípica por sexo.

## Nível da Coluna

Ao estratificar os níveis de acometimento da coluna por DS atípica ( Fig. 7 ), o nível de T10 a T12 foi o mais prevalente, representando 50% (n = 14) do total de casos positivos. Para a realização do teste ANOVA unidirecional e comparação de múltiplos grupos com o teste de contrastes lineares, os grupos de intervalo T9 a T11 e T8 a T10 foram combinados em um único grupo (T8–10/T9–T11). Houve uma diferença significativa no valor total do ângulo de Cobb entre os três grupos ( *p* = 0,02, tamanho do efeito = 0,68 [0,21–1,58]). Além disso, o teste de contrastes lineares detectou uma diferença significativa entre os grupos T10 a T12 e T11 a L1 ( *p* = 0,046). Por outro lado, a comparação de outros grupos não revelou nenhuma diferença significativa.

**Fig. 7:** Prevalência da doença de Scheuermann atípica por nível de acometimento da coluna.

## Escoliose

Ao comparar a presença de escoliose em pacientes com DS atípica de acordo com o sexo, não houve diferença significativa. A escoliose foi observada em 14% das mulheres e 25% dos homens (teste exato de Fisher, *p* = 1) ( Fig. 8 ).

**Fig. 8:** Comparação da presença de escoliose e sexo do paciente na doença de Scheuermann atípica

## Nódulos de Schmorl

Dos 28 pacientes com DS atípica, 10 (37%) apresentaram nódulos de Schmorl ao exame tomográfico ( Fig. 9 ). Não houve associação entre grupos etários ou sexo e a presença de nódulos de Schmorl ( *p* = 1).

**Fig. 9:** Presença de nódulos de Schmorl em pacientes com doença de Scheuermann atípica.

## Discussão

Neste estudo epidemiológico da população brasileira, encontramos uma prevalência de DS atípica de 2,8% utilizando os critérios definidos por Sorensen. 2

Como não existe um padrão uniforme para o diagnóstico radiológico da forma atípica da doença, optamos por utilizar os critérios de Sorensen por ser o mais utilizado em pesquisas anteriores. 15 20

O delineamento do nosso estudo, baseado em TCs, permitiu a identificação da DS atípica em pacientes sem queixas clínicas, que não teria sido detectada em uma análise baseada nas queixas clínicas do paciente. Como a procura pelo exame de tomografia foi devido a queixas que não relativas ao viés de seleção em nossa amostra foi menor, uma vez que a seleção de pacientes de um consultório de cirurgia de coluna incluiria a queixa de deformidade ou dor na coluna.

Tipicamente, os pacientes apresentam cifose de Scheuermann durante a fase de crescimento ósseo da adolescência. 5 7 8 Dessa forma, todos os pacientes que apresentaram as características radiológicas da doença já a tinham no momento da nossa avaliação. Além disso, para evitar a possibilidade de casos falso-positivos decorrentes de doença degenerativa do disco, limitamos os critérios de idade para inclusão dos pacientes em nosso estudo. Portanto, nenhum dos casos diagnosticados com DS atípica pode corresponder a pacientes com doença degenerativa do disco, e não deixamos de identificar nenhum paciente que ainda não tivesse desenvolvido a doença. Ainda há controvérsia na literatura quanto à prevalência da DS quanto ao sexo do paciente. Alguns estudos mostraram prevalência semelhante em ambos os sexos, 3 15 20 21 22 23 mas a maioria das pesquisas anteriores demonstrou uma incidência maior em homens. 1 3 8 17

No entanto, nenhum desses estudos avaliou separadamente pacientes diagnosticados com DS atípica. Assim, apesar de concordar com a maioria dos estudos, nossos resultados com maior prevalência de DS em homens representam apenas pacientes com a forma atípica da doença.

Sorensen descreveu critérios radiográficos para DS típica, incluindo cunha anterior maior que 5° em pelo menos três corpos vertebrais adjacentes. 2 Nódulos de Schmorl, placas terminais vertebrais irregulares, disco intervertebral estreito e alongamento anteroposterior dos corpos vertebrais apicais são outras características radiológicas associadas à DS. Por outro lado, Blumenthal et al. 24 descreveram os critérios da DS atípica, incluindo formação de cunha em um ou dois corpos vertebrais, alterações na placa terminal vertebral, estreitamento do espaço discal e nódulos de Schmorl anteriores.

Optamos por utilizar o critério de Sorensen de cunha vertebral anterior de mais de 5° porque ser mais objetivo do que simplesmente a cunha vertebral sugerida por Blumenthal et al. 24 Também pudemos investigar a presença de escoliose e nódulos de Schmorl e, assim como demonstrado em estudos anteriores, a escoliose foi observada em torno de um terço dos casos. 25 Em cerca de 40% dos exames, identificamos nódulos de Schmorl. Estes dados concordam com os achados de Heithoff et al., 12 que consideram a presença de nódulos de Schmorl como apenas um dos critérios diagnósticos de DS atípica.

A principal limitação do presente estudo reside no fato de termos utilizado o exame de TC para identificar a presença da DS, uma vez que o exame de escolha é a radiografia completa da coluna. No entanto, como o diagnóstico de DS atípica é baseado na formação de cunha em cada vértebra, o exame escolhido não afetou nossos resultados. Além disso, reduzimos a possibilidade de viés de seleção, uma vez que os pacientes foram submetidos ao exame por outras razões que não a coluna.

## Conclusão

A cifose de Scheuermann é uma deformidade espinhal frequente. Nosso estudo, obtido a partir de uma grande amostra de conveniência de pacientes avaliados com tomografia de abdome, permite uma melhor compreensão da real prevalência do padrão atípico desta doença. A TC permitiu estudar uma amostra populacional sem viés de seleção e encontrar uma prevalência de 2,8% de DS atípica dentre esses pacientes.
